# Stroke Subtype Among Individuals With Chronic Kidney Disease

**DOI:** 10.1177/20543581231203046

**Published:** 2023-10-14

**Authors:** Salmi T. Noor, Sarah E. Bota, Anna E. Clarke, William Petrcich, Dearbhla Kelly, Greg Knoll, Gregory L. Hundemer, Mark Canney, Peter Tanuseputro, Manish M. Sood

**Affiliations:** 1 Department of Medicine, The Ottawa Hospital, University of Ottawa, ON, Canada; 2 Ottawa Hospital Research Institute, ON, Canada; 3 Institute for Clinical Evaluative Sciences, Toronto, ON, Canada; 4 J. Philip Kistler Stroke Research Center, Massachusetts General Hospital, Harvard Medical School, Boston, USA

**Keywords:** stroke, CKD, dialysis, thromboembolic

## Abstract

**Background:**

It is widely accepted that there is a stepwise increase in the risk of acute ischemic stroke with chronic kidney disease (CKD). However, whether the risk of specific ischemic stroke subtypes varies with CKD remains unclear.

**Objective:**

To assess the association between ischemic stroke subtypes (cardioembolic, arterial, lacunar, and other) classified using the Trial of ORG 10172 in Acute Stroke Treatment (TOAST) and CKD stage.

**Design:**

retrospective cohort study.

**Setting:**

Ontario, Canada.

**Patients:**

A total of 17 434 adults with an acute ischemic stroke in Ontario, Canada between April 1, 2002 and March 31, 2013, with an estimated glomerular filtration rate (eGFR) measurement or receipt of maintenance dialysis captured in a stroke registry were included.

**Measurements:**

Kidney function categorized as an eGFR of ≥60, 30-59, <30 mL/min/1.73 m^2^, or maintenance dialysis. Ischemic stroke classified by TOAST included arterial, cardioembolic, lacunar, and other (dissection, prothrombotic state, cortical vein/sinus thrombosis, and vasculitis) types of strokes.

**Methods:**

Adjusted regression models.

**Results:**

In our cohort, 58.9% had an eGFR of ≥60, 34.7% an eGFR of 30-59, 6.0% an eGFR of <30 and 0.5% were on maintenance dialysis (mean age of 73 years; 48% women). Cardioembolic stroke was more common in patients with non-dialysis-dependant CKD (eGFR 30-59: 50.4%, adjusted odds ratio [OR] 1.20, 95% confidence interval [CI]: 1.02, 1.44; eGFR<30: 50.6%, OR 1.21, 95% CI: 1.02, 1.44), whereas lacunar stroke was less common (eGFR 30-59: 22.7% OR 0.85, 95% CI: 0.77, 0.93; eGFR <30: 0.73, 95% CI: 0.61, 0.88) compared with those with an eGFR ≥60. In stratified analyses by age and CKD, lacunar strokes were more frequent in those aged less than 65 years, whereas cardioembolic was higher in those aged 65 years and above.

**Limitations:**

TOAST classification was not captured for all patients.

**Conclusion:**

Non-dialysis CKD was associated with a higher risk of cardioembolic stroke, whereas an eGFR ≥60 mL/min/1.73 m^2^ was associated with a higher risk of lacunar stroke. Detailed stroke subtyping in CKD may therefore provide mechanistic insights and refocus treatment strategies in this high-risk population.

## Abrégé

**Contexte:**

Il est largement admis qu’il y a une augmentation progressive du risque d’accident vasculaire cérébral ischémique aigu en contexte d’insuffisance rénale chronique (IRC). On ignore cependant si le risque de certains sous-types particuliers d’AVC ischémiques varie en présence d’IRC.

**Objectif:**

Évaluer le lien entre le stade d’IRC et certains sous-types d’AVC ischémiques (cardioembolique, artériel, lacunaire et autres) classés selon l’essai TOAST (Trial of ORG 10172 in Acute Stroke Treatment).

**Type d’étude:**

Étude de cohorte retrospective.

**Sujets:**

Ont été inclus 17 434 adultes ayant subi un AVC ischémique aigu en Ontario (Canada) entre le 1er avril 2002 et le 31 mars 2013, et pour lesquels le registre d’AVC comportait une mesure du débit de filtration glomérulaire estimé (DFGe) ou une dialyze chronique.

**Mesures:**

La fonction rénale a été classée selon le DFGe (≥ 60 ml/min/1,73 m^2^ **—**entre 30 et 59 ml/min/1,73 m^2^ **—**<30 ml/min/1.73 m^2^) ou une dialyze chronique. Les types d’AVC ischémiques classés par l’essai TOAST comprenaient les AVC artériels, cardioemboliques, lacunaires et autres (dissection, état prothrombotique, thrombose de la veine/sinus cortical, vascularite).

**Méthodologie:**

Modèles de régression ajustés.

**Résultats:**

Dans notre cohorte (âge moyen de 73 ans; 48% de femmes), 58,9 % des patients avaient un DFGe ≥ 60 ml/min/1,73 m^2^; 34,7% avaient un DFGe entre 30 et 59 ml/min/1,73 m^2^; 6,0 % avaient un DFGe < 30 ml/min/1,73 m^2^ et 0,5 % des patients étaient en dialyze chronique En comparaison des patients ayant un DFGe ≥ 60 ml/min/1,73 m^2^, les AVC cardioemboliques étaient plus fréquents chez les patients atteints d’IRC sans dialyze (DFGe entre 30 et 59 ml/min/1,73 m^2:^ 50,4%; rapport de cote corrigé [RCc] = 1,20; IC 95 % = 1,02-1,44**—**DFGe < 30 ml/min/1,73 m^2:^ 50,6 %; RCc = 1,21; IC95% = 1,02-1,44) alors que les AVC lacunaires étaient moins fréquents [DFGe entre 30 et 59 ml/min/1,73 m^2:^ 22,7%; RCc = 0,85; IC 95% = 0,77-0,93**—**DFGe < 30 ml/min/1,73 m^2:^ RCc = 0,73; IC 95% = 0,61-0,88]. Dans les analyses stratifiées en fonction de l’âge et de l’IRC, les AVC lacunaires étaient plus fréquents chez les moins de 65 ans tandis que les AVC cardioemboliques étaient plus fréquents chez les plus de 65 ans.

**Limites:**

La classification TOAST n’était pas enregistrée pour tous les patients.

**Conclusion:**

L’IRC sans dialyze a été associée à un risque plus élevé d’AVC cardioembolique alors qu’un DFGe ≥ 60 ml/min/1.73 m^2^ a été associé à un risque plus élevé d’AVC lacunaire. Le sous-typage détaillé des AVC en contexte d’IRC pourrait donc fournir des informations mécanistiques et recentrer les stratégies de traitement dans cette population à haut risque.

## What was known before

- Individuals with chronic kidney disease (CKD) or on long-term hemodialysis are at an exceedingly high risk of ischemic stroke. Little is known regarding the anatomic details delineating the stroke type.

## What this adds

- Using data from 17 434 adults with an ischemic stroke, cardioembolic stroke was more common in patients with non-dialysis-dependant CKD, whereas lacunar stroke was less common compared with those with an estimated glomerular filtration rate (eGFR) ≥60.

## What impact this may have on practice or policy

- Understanding detailed stroke subtyping in CKD may provide mechanistic insights and refocus treatment strategies in this high-risk population.

## Introduction

Stroke affects approximately 24.9% of people aged 25 years or older, worldwide.^1^ Although incidence rates remain stable, this prevalence is increasing as afflicted individuals are living longer.^2,3^ Stroke is particularly common in patients with chronic kidney disease (CKD).^4,5^ In a systematic review by Masson et al,^6^ a glomerular filtration rate (GFR) <90 mL/min/1.73 m^2^ was associated with a risk of all-cause stroke by 39%, with a stepwise increase in stroke risk with declining renal function. Stroke and CKD share several common vascular risk factors, such as atrial fibrillation (AF), hypertension, atherosclerosis, and diabetes, all of which are known to result in poor long-term health outcomes.^6,7^ Although studies report the association between all-cause stroke in patients and CKD, little is known regarding the underlying etiologic stroke type and how it differs in CKD.^6,8,9^

Strokes are broadly classified as ischemic or hemorrhagic.^10^ Several guidelines exist for the categorization of ischemic strokes, including the ASCOD phenotyping system (atherosclerosis, small-vessel disease, cardiac pathology, and other causes) and the Causative Classification System, but the most frequently used tool is the Trial of ORG 10172 in Acute Stroke Treatment (TOAST) classification system.^8^,^10-12^ The TOAST system categorizes ischemic strokes into one of five domains: (1) large artery atherosclerosis, (2) cardioembolic (CE), (3) small-vessel occlusion, (4) stroke of other determined etiology, and (5) stroke of undetermined etiology.^10^

To date, several randomized control trials provide evidence on the relationship between CKD and ischemic stroke, with CKD being one of the largest risk factors of stroke.^6,7^ However, few report subtype-specific associations and how these subtypes vary according to CKD stage and severity. Therefore, we examined different ischemic stroke subtypes, captured in a validated stroke registry, in adult patients with CKD and maintenance dialysis in Ontario, Canada.

## Methods

### Design, Setting, and Data Sources

We conducted a cohort study of adults (aged 18 years or older) with a known estimated glomerular filtration rate (eGFR) measure or receiving maintenance dialysis therapy who presented to the emergency department or were hospitalized with an acute stroke using de-identified, linked databases available at ICES (formerly the Institute for Clinical Evaluative Sciences). Stroke etiology and patient health data from all hospitalizations were determined using the Ontario Stroke Registry (OSR). The OSR is a provincial registry that collected data on all stroke patients cared for at a Regional Stroke Center and a population-based sample of patients cared for at other acute hospitals.^13^ Baseline characteristics, clinical outcomes and stroke subtype, and kidney function were identified using the OSR. In addition, data on patient demographics and eGFR were determined using the RPDB (Registered Persons Database) and OLIS (The Ontario Laboratory Information System), respectively. These data sets were linked using unique encoded identifiers and analyzed at ICES. The reporting of this study follows the RECORD (REporting of studies Conducted using Observational Routinely collected Data) guidelines for observational studies (see Supplemental Table 3).^14^ Whenever possible, we defined patient characteristics and outcomes using codes from the OSR, which were collected using chart abstraction by trained stroke abstractors (see Supplemental Table 2). The use of data in this project was authorized under section 45 of Ontario’s Personal Health Information Protection Act, which does not require review by a research ethics board.

### Study Population

All patients with a diagnosis of ischemic stroke at discharge from April 1, 2002, to March 31, 2013, in Ontario, Canada (see Supplemental Figure 1 for cohort creation) were included (n=42 307). Exclusion criteria were (1) age <18 years (n=78), (2) missing inpatient serum creatinine (n=6344), (3) evidence of an in-hospital stroke (n=785), (4) missing stroke etiology (n=17 486), and (5) history of a kidney transplant (n=33). Patients with missing or invalid age were also excluded. After data cleaning, and linking of administrative data sets, the exclusion criteria was applied and a total of 17 434 patients were included in the final analysis.

### Exposure and Covariates

The main study exposure was eGFR or maintenance dialysis with eGFR categorized as follows: ≥60, 30-59, and <30 mL/min/1.73 m^2^, or on maintenance dialysis (hemodialysis and peritoneal dialysis).^15^ The OSR was used to obtain baseline eGFR and it was determined using the serum creatinine measure first available upon presentation to the ED and calculated using the CKD-EPI equation.^16^ The study index date was the date of arrival to hospital for the stroke event. Baseline characteristics such as age, sex, year, income quintile, rurality, and pre-event residence were all collected at index date. Information was also collected on patients’ medical conditions prior to index, such as diabetes, hypertension, hyperlipidemia, cigarette smoking history, stroke, major hemorrhage, transient ischemic attack, congestive heart failure/pulmonary edema, peripheral vascular disease, AF or flutter, valvular heart disease, valve replacement, venous thromboembolism, carotid endarterectomy or stenting, coronary artery disease, revascularization, active cancer, cirrhosis, systemic hemorrhage (peptic ulcer disease, gastrointestinal bleed), and the Charlson Comorbidity Index. Both international normalized ratio (INR; at hospital presentation) and albumin-to-creatinine ratio (ACR; within 1 year) were obtained along with anticoagulant, antiplatelet, and warfarin use.

### Outcomes

The study outcome was an ischemic stroke, categorized as one of the following: large artery atherosclerosis, CE, lacunar, or other (dissection, prothrombotic state, cortical vein/sinus thrombosis, vasculitis, or other; see Supplemental Table 2 for full data definitions).

### Statistical Analysis

Categorical data are presented as n (%) and continuous data are presented in the form of medians (interquartile range [IQR]). We calculated the crude frequency for every stroke type overall and by eGFR category. Crude and adjusted prevalence odds ratios (ORs) were estimated using multivariable logistic regression. We created separate adjusted models examining the association of eGFR categories (eGFR≥30, eGFR <30, and dialysis) with each stroke type (CE, lacunar, arterial, and other). Variables in the partially adjusted models included the following: age, sex, index year hypertension, and diabetes. This was performed using staggered adjustment, where first only age, sex, and index year were included, and hypertension and diabetes were added afterward to explore whether the direction and magnitude of associations changed significantly. We further described stroke type stratified by key groups of age (> or <65 years), ACR (<3 or ≥3 mg/mmol), presence of AF and among those with AF, and receipt of anticoagulation. Analyses were conducted with SAS Enterprise Guide, Version 7.1 (SAS Institute Inc., Cary, North Carolina).

## Results

A total of 17 434 patients were included in the study cohort. Table 1 shows the baseline characteristics of all patients at the time of, and prior to, the stroke event according to eGFR category and maintenance dialysis type. The mean age was 75 (64-83) years, 48% (n=8368) were women, and with hypertension present in 71% of patients (n=12 332). Of the entire cohort, 10 266 (59%) had an eGFR ≥60, 6044 (35%) an eGFR 30-59, and 1041 (6.0%) an eGFR<30. A total of 83 patients were on maintenance dialysis, with 67 hemodialysis and 16 peritoneal dialysis patients. Patients with an eGFR <30 were older, with a mean age of 82 (72-88) years. They were also more commonly women (58%) and had a higher likelihood of comorbidities compared with those with an eGFR >30. Most patients had an INR category <1.2 (73%) and the median ACR was 4 (IQR 2-16). A total of 10% were on anticoagulants, with the majority on warfarin, and 29% were on antiplatelets.

**Table 1.** Baseline Characteristics by Estimated Glomerular Filtration Rate (eGFR) Category.

Characteristic	Total N=17 434	High, normal, and mildly decreased ≥60 mL/min/1.73 m^2^ N=10 266	Moderately decreased 30-59 mL/min/1.73 m^2^ N=6044	Severely decreased and kidney failure <30 mL/min/1.73 m^2^ N=1041	Maintenance dialysis N=83
Demographics					
Age (years)					
Median (IQR)	75 (64-83)	70 (59-79)	81 (74-86)	82 (76-88)	71 (61-77)
Sex, female	8368 (48.0%)	4338 (42.3%)	3396 (56.2%)	606 (58.2%)	28 (33.7%)
Comorbidities					
Diabetes	4481 (25.7%)	2462 (24.0%)	1572 (26.0%)	406 (39.0%)	41 (49.4%)
Hypertension	12 332 (70.7%)	6586 (64.2%)	4806 (79.5%)	865 (83.1%)	75 (90.4%)
Hyperlipidemia	6866 (39.4%)	3832 (37.3%)	2517 (41.6%)	472 (45.3%)	45 (54.2%)
History of smoking					
Current nonsmoker	3809 (21.8%)	2130 (20.7%)	1380 (22.8%)	276 (26.5%)	23 (27.7%)
Current smoker	2833 (16.2%)	2157 (21.0%)	591 (9.8%)	76 (7.3%)	9 (10.8%)
Former smoker	1286 (7.4%)	757 (7.4%)	426 (7.0%)	90-94 (8.6%-9.0%)	9-13 (10.9%-15.7%)
Lifelong nonsmoker	1011 (5.8%)	613 (6.0%)	333 (5.5%)	60-64 (5.8%-6.1%)	1-5(1.2%-6.0%)
Stroke	3322 (19.1%)	1655 (16.1%)	1359 (22.5%)	284 (27.3%)	24 (28.9%)
Transient ischemic attack	2298 (13.2%)	1232 (12.0%)	877 (14.5%)	177 (17.0%)	12 (14.5%)
Congestive heart failure or pulmonary edema	1779 (10.2%)	643 (6.3%)	877 (14.5%)	242 (23.2%)	17 (20.5%)
Peripheral vascular disease	1048 (6.0%)	483 (4.7%)	424 (7.0%)	117 (11.2%)	24 (28.9%)
Atrial fibrillation or flutter	4520 (25.9%)	2087 (20.3%)	2074 (34.3%)	340 (32.7%)	19 (22.9%)
Valvular heart disease	920 (5.3%)	472 (4.6%)	363 (6.0%)	79 (7.6%)	6 (7.2%)
Heart valve replacement	427 (2.4%)	245 (2.4%)	151 (2.5%)	23-28 (2.2%-2.7%)	1-5 (1.2%-6.0%)
Deep vein thrombosis or pulmonary embolism	479 (2.7%)	245 (2.4%)	192 (3.2%)	34-39 (3.3%-3.7%)	1-5 (1.2%-6.0%)
Carotid endarterectomy or stenting	284 (1.6%)	129 (1.3%)	119 (2.0%)	30 (2.9%)	6 (7.2%)
Coronary artery disease^a^	4339 (24.9%)	2106 (20.5%)	1809 (29.9%)	382 (36.7%)	42 (50.6%)
Cancer	1506 (8.6%)	788 (7.7%)	595 (9.8%)	115 (11.0%)	8 (9.6%)
Cirrhosis of the liver	71 (0.4%)	38 (0.4%)	24 (0.4%)	3-8 (0.3%-0.8%)	1-5 (1.2%-6.0%)
Systemic hemorrhage	117 (0.7%)	39 (0.4%)	62 (1.0%)	16 (1.5%)	0 (0.0%)
Charlson comorbidity index					
Median (IQR)	1 (0-3)	1 (0-2)	1 (0-3)	3 (1-4)	4 (3-6)
Laboratory measurements					
International normalized ratio (INR)					
0.0-<1.2	12 756 (73.2%)	7802 (76.0%)	4231 (70.0%)	678 (65.1%)	45 (54.2%)
1.2-<2.0	2602 (14.9%)	1287 (12.5%)	1074 (17.8%)	223 (21.4%)	18 (21.7%)
2.0-<3.0	536 (3.1%)	256 (2.5%)	241 (4.0%)	32-37 (3.1%-3.6%)	1-5 (1.2%-6.0%)
3.0-4.5	150 (0.9%)	67 (0.7%)	70 (1.2%)	7-12 (0.7%-1.2%)	1-5 (1.2%-6.0%)
4.5+	48 (0.3%)	18 (0.2%)	20 (0.3%)	4-9 (0.4%-0.9%)	1-5 (1.2%-6.0%)
Albumin-to-creatinine ratio (ACR)					
Median (IQR)	4 (2-16)	3 (1-9)	5 (2-17)	17 (4-94)	154 (118-206)
Medications					
Anticoagulant	1765 (10.1%)	820 (8.0%)	795 (13.2%)	136 (13.1%)	14 (16.9%)
Antiplatelet	4978 (28.6%)	2643 (25.7%)	1950 (32.3%)	348 (33.4%)	37 (44.6%)
Warfarin	1593 (9.1%)	716 (7.0%)	741 (12.3%)	125 (12.0%)	11 (13.3%)

*Note*. In accordance with ICES privacy policies, cell sizes less than or equal to 5 cannot be reported. IQR = interquartile range.

a Coronary artery disease definition includes myocardial infarction, angina, percutaneous coronary intervention, and coronary artery bypass graft.

The most common type of ischemic stroke was CE (42%) followed by lacunar (25%), arterial (21%), and other (11%). This was consistent when stratified by eGFR/dialysis categories (Table 2). When examining stroke type by eGFR, eGFR categories of ≥30 and <30 were both associated with a higher adjusted OR of CE stroke (eGFR ≥30: OR 1.33, 95% CI: 1.23, 1.43; eGFR<30: OR 1.30, 95% CI: 1.13, 1.49) compared with an eGFR ≥60 (referent group). Conversely, both eGFR categories of ≥30 and <30 were associated with a lower OR of lacunar stroke (eGFR ≥30: OR 0.75, 95% CI: 0.69, 0.82; eGFR <30: OR 0.68, 95% CI: 0.58, 0.80) compared with an eGFR ≥60. No significant association by eGFR category and arterial or other stroke type was observed.

**Table 2.** Ischemic Stroke Type by Estimated Glomerular Filtration Rate (eGFR) Category.

Stroke type	Total	High, normal, and mildly decreased ≥60 mL/min/1.73 m^2^	Moderately decreased 30-59 mL/min/1.73 m^2^	Severely decreased and kidney failure <30 mL/min/1.73 m^2^	Maintenance dialysis
n	17 434	10 266	6044	1041	83
Large artery atherosclerosis	3703 (21.2%)	2290 (22.3%)	1182 (19.6%)	212 (20.4%)	19 (22.9%)
Cardioembolic	7386 (42.4%)	3780 (36.8%)	3048 (50.4%)	527 (50.6%)	31 (37.3%)
Lacunar	4425 (25.4%)	2805 (27.3%)	1369 (22.7%)	226 (21.7%)	25 (30.1%)
Other^a^	1920 (11.0%)	1391 (13.5%)	445 (7.4%)	76 (7.3%)	8 (9.6%)

*Note*. In accordance with ICES privacy policies, cell sizes less than or equal to 5 cannot be reported. ICES = formerly the Institute for Clinical Evaluative Sciences.

a Other includes dissection, prothrombotic state, cortical vein/sinus thrombosis, vasculitis, and other stroke types.

In individuals aged <65 and 65+ years, there were significant differences in stroke types (*p* values < .001 and .03, respectively; Table 3). Based on eGFR <30 or dialysis and eGFR 30 or greater, significant differences were found in both CE and lacunar stroke types (*p* values .016 and .006, respectively). Significant differences in stroke type were found among individuals with ACR <3 (*p* value .03), whereas for those with ACR >3, findings were nonsignificant (*p* value .73). Among individuals with AF, no significant differences were found between stroke types and eGFR subgroups. However, in individuals with no AF, there were significant differences between stroke types (*p* value <.001) and specifically for CE stroke (*p* value <.001) when comparing eGFR subgroups.

**Table 3.** Stroke Type by Age (<65, 65+ years), Albuminuria (< 3, 3+ mmol/mg), Atrial Fibrillation, and Anticoagulation Therapy.

	Stroke type	Total	eGFR <30 or dialysis	eGFR 30 or greater	*P* value
		N=4406	N=103	N=4303	<.001^a^
Age <65 years	Cardioembolic	1184 (26.9)	35 (34.0)	1149 (26.7)	.1
	Lacunar	1260 (28.6)	39 (37.9)	1221 (28.4)	.035
	Artery	1026 (23.3)	23 (22.3)	1003 (23.3)	.816
	Other	936 (21.2)	6 (5.8)	903 (21.6)	<.001^b^
Age 65+ years		N=13 028	N=1021	N=12 007	.03^a^
	Cardioembolic	6202 (47.6)	523 (51.2)	5679 (47.3)	.016^b^
	Lacunar	3165 (24.3)	212 (20.8)	2953 (24.6)	.006^b^
	Artery	2677 (20.5)	208 (20.4)	2469 (20.6)	.885
	Other	984 (7.6)	78 (7.6)	906 (7.5)	.913
ACR <3		N=447	N=25	N=422	.73^c^
	Cardioembolic	223 (49.9%)	15 (60.0%)	208 (49.3%)	.298
	Lacunar	100 (22.4%)	1-5 (4%-20.0%)	91-96 (21.6%-22.7%)	.431
	Artery	95 (21.3%)	1-5 (4%-20.0%)	85-90 (20.1%-21.3%)	.875
	Other	29 (6.5%)	1-5 (4%-20.0%)	23-28 (5.5%-6.6%)	.603
ACR ≥3		N=534	N=83	N=451	.91^c^
	Cardioembolic	292 (54.7%)	46 (55.4%)	246 (54.5%)	.883
	Lacunar	111 (20.8%)	17 (20.5%)	94 (20.8%)	.941
	Artery	106 (19.9%)	10-15 (12.4%-18.1%)	91-96 (20.2%-21.3%)	.659
	Other	25 (4.7%)	1-5 (1.2%-6.0%)	15-20 (3.3%-4.4%)	.659
Atrial fibrillation (yes)		N=4520	N=359	N=4161	.52^c^
	Cardioembolic	3828 (84.7%)	307 (85.5%)	3521 (84.6%)	.651
	Lacunar	264 (5.8%)	17 (4.7%)	247 (5.9%)	.352
	Artery	293 (6.5%)	27 (7.5%)	266 (6.4%)	.405
	Other	135 (3.0%)	27 (7.5%)	127 (3.1%)	.379
Atrial fibrillation (no)		N=12 914	N=765	N=12 149	<.001^a^
	Cardioembolic	3558 (27.6%)	251 (32.8%)	3307 (27.2%)	<.001^b^
	Lacunar	4161 (32.2%)	234 (30.6%)	3927 (32.3%)	.319
	Artery	3410 (26.4%)	204 (26.7%)	3206 (26.4%)	.866
	Other	1785 (13.8%)	76 (9.9%)	1709 (14.1%)	.001^b^
Among atrial fibrillation, anticoagulation = Yes		N=1391	N=105	N=1286	.07^c^
	Cardioembolic	1142 (82.1%)	94 (89.5%)	1048 (81.5%)	.039^b^
	Lacunar	110 (7.9%)	1-5 (1.0%-4.8%)	102-107 (7.9%-8.3%)	.046^b^
	Artery	83 (6.0%)	7 (6.7%)	76 (5.9%)	.753
	Other	56 (4.0%)	1-5 (1.0%-4.8%)	50-55 (3.9%-4.3%)	.096
Among atrial fibrillation, anticoagulation = No		N=3129	N=254	N=2875	.82^c^
	Cardioembolic	2686 (85.8%)	213 (83.9%)	2473 (86.0%)	.344
	Lacunar	154 (4.9%)	14 (5.5%)	140 (4.9%)	.65
	Artery	210 (6.7%)	20 (7.9%)	190 (6.6%)	.44
	Other	79 (2.5%)	7 (2.8%)	72 (2.5%)	.806

*Note*. Fields without annotation show no significant difference in stroke types based on eGFR subgroups (eGFR <30 or dialysis and eGFR 30 or greater). In accordance with ICES privacy policies, cell sizes less than or equal to 5 cannot be reported. eGFR = estimated glomerular filtration rate; ACR = albumin-to-creatinine ratio; ICES = formerly the Institute for Clinical Evaluative Sciences.

a Significant difference in stroke types.

b Significant difference in stroke types based on eGFR subgroups (eGFR <30 or dialysis and eGFR 30 or greater).

c No significant differences in stroke types.

After adjusting for age, sex, index year, income quintile, comorbidities, Charlson score, and medications (antiplatelets/anticoagulants), the odds of having a CE stroke was higher compared with the odds of having arterial or lacunar strokes across both eGFR categories, however within CE stroke, there were no significant differences between eGFR categories predicting these strokes; eGFR <30: OR 1.21, 95% CI: 1.02, 1.44 and eGFR ≥30: OR 1.20, 95% CI: 1.10, 1.31 (Table 4).

**Table 4.** Adjusted OR (95% Confidence Intervals) of Stroke Type by eGFR Category.

Stroke type	Kidney function		
	eGFR ≥30	eGFR <30	Maintenance dialysis
Artery	0.85 (0.78, 0.92)	0.89 (0.76, 1.04)	1.03 (0.62, 1.73)
Lacunar	0.78 (0.72, 0.84)	0.74 (0.63, 0.86)	1.15 (0.72, 1.84)
CE	1.75 (1.64, 1.86)	1.76 (1.55, 2.00)	1.02 (0.65, 1.60)
Other	0.51 (0.45, 0.57)	0.50 (0.40, 0.64)	0.68 (0.33, 1.41)
Adjusted for age, sex, and index year			
Artery	0.95 (0.87, 1.03)	1.01 (0.86, 1.19)	1.00 (0.59, 1.67)
Lacunar	0.78 (0.72, 0.85)	0.75 (0.64, 0.89)	1.21 (0.75, 1.95)
CE	1.29 (1.20, 1.39)	1.22 (1.06, 1.40)	0.97 (0.61, 1.53)
Other	0.90 (0.79, 1.02)	0.93 (0.72, 1.19)	0.87 (0.41, 1.83)
Adjusted for age, sex, index year, hypertension, and diabetes			
Artery	0.93 (0.85, 1.01)	0.98 (0.83, 1.16)	0.94 (0.56, 1.57)
Lacunar	0.75 (0.69, 0.82)	0.68 (0.58, 0.80)	1.03 (0.64, 1.67)
CE	1.33 (1.23, 1.43)	1.30 (1.13, 1.49)	1.06 (0.67, 1.69)
Other	0.94 (0.83, 1.06)	1.02 (0.79, 1.31)	1.04 (0.50, 2.19)
Adjusted for age, sex, index year, income quintile, comorbidities, Charlson score, and medications (antiplatelet, anticoagulants)			
Artery	0.95 (0.86, 1.04)	1.01 (0.84, 1.21)	1.02 (0.55, 1.89)
Lacunar	0.85 (0.77, 0.93)	0.73 (0.61, 0.88)	1.25 (0.68, 2.28)
CE	1.20 (1.10, 1.31)	1.21 (1.02, 1.44)	0.86 (0.48, 1.57)
Other	1.00 (0.88, 1.15)	1.11 (0.84, 1.46)	1.10 (0.46, 2.66)

*Note*. OR = odds ratio; eGFR = estimated glomerular filtration rate; CE = cardioembolic.

## Discussion

Using a province-based stroke registry of 17 434 individuals presenting with an acute ischemic stroke and available kidney function parameters, we found that stroke types differed by eGFR stages. Cardioembolic stroke was more frequently associated with non-dialysis-dependent CKD, with an eGFR below 60 mL/min, whereas lacunar stroke was less frequent. When examining pertinent risk factors, CE strokes were more common among individuals aged 65 years or older, with more advanced stages of CKD among those without a history of AF and among those with AF on anticoagulation compared with those with less advanced CKD.

Our findings are consistent with and expand on previous studies finding CE stroke to be the most common stroke with lower eGFR.^6,17,18^ Sozio et al^19^ reported CE stroke to be the most common (accounting for 28%) stroke type among incident dialysis patients. Examining individuals with less severe CKD (mean eGFR 47 mL/min), Kelly et al reported a higher prevalence of CE strokes in 1197 patients with CKD.^8^ Our study, including more than 7000 individuals with CKD and using high-quality data from a stroke registry, further demonstrates that CE strokes are the predominant stroke type in the CKD population.

Among those with a history of AF, CE strokes were the dominant stroke type, accounting for nearly 85% of all stroke types across all stages of CKD, and reemphasize the importance of determining the optimal treatment strategy for this population.^18,20,21^ Interestingly, we found no significant difference in CE strokes by anticoagulation status, suggesting that treatment failures may be common. With warfarin use, subtherapeutic and supratherapeutic INR levels occur frequently among the CKD/dialysis population and, even among trial settings, the ability to maintain a time in the therapeutic range is often below 40%.^22-24^

Cardioembolic strokes are linked to adverse outcomes in terms of functional recovery and mortality.^25,26^ In general, they carry a poor prognosis shown by early and long-term stroke recurrence as well as high in-hospital mortality compared with other subtypes.^27^ Individuals on long-term dialysis with a CE stroke tend to have high fatality and low recovery rates, with a 30-day mortality rate of 18% and a 1-year mortality rate higher than 50%.^9,28^ Detection may also be delayed in dialysis patients as they often present later after symptom onset, limiting health care interventions.^28,29^

Interestingly, we found that severely and moderately decreased GFR had an inverse relationship with the development of lacunar strokes. Previous studies have reported silent lacunar infarcts to be common, often clinically silent, and accrue over time on dialysis.^8^,^30-33^ Our cohort may represent a selection bias of more severe stroke types, who present with clinical symptoms and may under-represent the true totality of lacunar small-vessel infarcts, many of which are identified coincidentally on imaging.^34^

Studies have suggested that common risk factors, such as hypertension and diabetes, are not particularly associated with lacunar strokes and arise primarily from classification bias.^31^ Misclassification may be further magnified in the CKD/kidney failure population as they are less likely to get appropriate investigations such as magnetic resonance imagings to detect lacunar strokes.^35-37^ Moreover, patients who present with lacunar strokes can often be prone to multiple subtypes of strokes, which further increases potential misclassification.^38,39^

The findings of this study hold broader significance for future stroke and CKD research. While it is important to know the associations between stroke and declining kidney function, it becomes even more important to understand associations between different stroke subtypes. Understanding subtype-specific associations and how they contribute to the classification of different stroke subtypes ultimately forms the basis to inform patient treatment, management, and prognosis. Our study sheds light on this classification and facilitates further exploration of these individual relationships.

Our study also has several limitations. First, while arterial, CE, and lacunar strokes were explored in this study, “other stroke” occurred in 11%, with no further subclassification.^8^ Second, the findings in this study cannot establish causality due to it being a retrospective, population-based cross-sectional study. Third, this study used a classification system to categorize stroke subtypes, and classification systems have several of their own shortcomings.^8,40^ While the TOAST classification system is the most widely used system, it is possible that other pathways and subtypes of stroke are not adequately captured in this system. Fourth, our study used one creatinine value and it is likely this contributed to some level of misclassification in determining acute versus stable kidney function.^41^ Fifth, the stroke registry would tend to capture more severe events and possibly miss clinically silent strokes, a phenomenon that is more common in those with CKD. Finally, our cohort included a small proportion of individuals with ischemic strokes, which may affect generalizability. However, given that our research aims to provide a descriptive foundation for the relationship between CKD and stroke in the province, we feel that findings are likely to still follow similar direction of associations, and future research can build on generalizable findings in this population.

In summary, stroke subtypes using the TOAST classification differed by kidney function, with CE and lacunar infarcts occurring more and less frequently, respectively, among those with moderately or severely decreased eGFR. Further studies focusing on the patient experience and clinically relevant outcomes by stroke type in individuals with advanced CKD would aid in prognostication and management.

## Supplemental Material

sj-docx-1-cjk-10.1177_20543581231203046 – Supplemental material for Stroke Subtype Among Individuals With Chronic Kidney Disease Click here for additional data file. Supplemental material, sj-docx-1-cjk-10.1177_20543581231203046 for Stroke Subtype Among Individuals With Chronic Kidney Disease by Salmi T. Noor, Sarah E. Bota, Anna E. Clarke, William Petrcich, Dearbhla Kelly, Greg Knoll, Gregory L. Hundemer, Mark Canney, Peter Tanuseputro and Manish M. Sood in Canadian Journal of Kidney Health and Disease
