# Supplementary material for: Stroke Subtype Among Individuals With Chronic Kidney Disease
Source: Can J Kidney Health Dis. 2023 Oct 14;10:20543581231203046. doi: 10.1177/20543581231203046 (PMC10576427; doi:10.1177/20543581231203046)
Supplement: sj-docx-1-cjk-10.1177_20543581231203046 – Supplemental material for Stroke Subtype Among Individuals With Chronic Kidney Disease [file sj-docx-1-cjk-10.1177_20543581231203046.docx]

**Data Supplement**

**Supplemental Figure 1.** Cohort creation flow diagram

**Supplemental Table 1.** Description of ICES databases used in this study

**Supplemental Table 2.** Administrative data definitions for inclusion, exposure and outcomes included in this study

**Supplemental Table 3.** Reporting of studies Conducted using Observational Routinely collected health Data (RECORD) statement checklist

**Supplemental Table 4.** Description of TOAST Classifications

**Supplemental Figure 1.** Cohort creation flow diagram

**Supplemental Table 1.** Description of ICES databases used in this study

| **Database** | **Description** |
| --- | --- |
| **Ontario Stroke Registry (OSR)** | The OSR captures suspected acute stroke and transient ischemic attacks seen in an emergency department of admitted to a hospital in Ontario. Data was collected consecutively at the Regional Stroke Centres between July 1, 2003, and March 31, 2013, and a population-based sample of patients seen at the other acute care facilities in fiscal years 2002, 2004, 2008, 2010, and 2012. The database includes information on demographics, stroke characteristics, comorbid conditions, medications, investigations, consultations, treatments, and in-hospital outcomes. |
| **Canadian Institute of Health Information Discharge Abstract Database (CIHI-DAD)** | The CIHI-DAD collects diagnostic, and procedural variables for each admission to a hospital in Ontario. Coding of primary and secondary diagnoses and inpatient procedures uses the 9th version of the Canadian Modified International Classification of Disease system (ICD-9 CA) prior to 2002 and the 10th version (ICD-10 CA) for all diagnoses after 2002. We will use the CIHI-DAD to obtain demographics, assess hospitalizations prior to the index date and co-morbid conditions for each patient in the five years prior to the index date. These characteristics act as study inclusion or exclusion criteria, or confounders in the multivariable models. |
| **Ontario Health Insurance Plan (OHIP) Claims History Database** | Most physicians in Ontario submit billing claims using fee and diagnosis codes outlined in the OHIP Schedule of Benefits. These codes capture information on inpatient, outpatient, and laboratory services rendered to a patient. In addition, OHIP includes information on the nature of the service and diagnostic information. In chart re-abstraction studies, agreement between abstracted OHIP codes compared to the actual code recorded on the chart by the physician for the “most responsible” diagnosis was over 90% while percent agreement for procedural codes was over 88%. |
| **Registered Persons Database (RPDB**) | The RPDB captures information regarding Ontarians’ gender, date of birth, postal code, and vital status. |
| **Canadian Organ Replacement Register (CORR)** | CORR is a national information system which records and analyzes the level of activity and outcomes of vital organ transplantation and dialysis activities. |
| **National Ambulatory Care Reporting System (NACRS)** | The NACRS is compiled by the Canadian Institute for Health Information (CIHI) and contains administrative, clinical (diagnoses and procedures), demographic, and administrative information for all patient visits made to hospital- and community-based ambulatory care centers (emergency departments, day surgery units, hemodialysis units, and cancer care clinics) in Ontario. At ICES, NACRS records are linked with other data sources (DAD, Ontario Mental Health Reporting System [OMHRS]) to identify transitions to other care settings, such as inpatient acute care or psychiatric care. Prior to April 1, 2002, diagnoses (up to 6 on a given NACRS record) are captured using the ICD-9 coding system and procedures (up to 10 on a given NACRS record) are captured using the CCP coding system. Following April 1, 2002, diagnoses (up to 10 on a given NACRS record) are captured using the ICD-10-CA coding system and interventions (up to 10 on a given NACRS record) are captured using the CCI coding system. NACRS emergency department diagnosis codes have been extensively validated. |
| **Ontario Laboratory Information System (OLIS)** | The Ontario Laboratory Information System (OLIS) is an electronic system that contains laboratory tests conducted for patients in Ontario. Data is available from 2007 to 2016 with serum creatinine values cleaned and at ICES Central. In the database the number of individuals older than 66 years having at least one serum creatinine is greater than 3 million. This database will allow us to establish a subset of patients with chronic kidney disease defined by serum creatinine laboratory values and estimated glomerular filtration rates. |

**Supplemental Table 2.**Administrative data definitions for inclusion, exposure and outcomes included in this study

| **Variable** | **Database** | **Details** | **Codes** |
| --- | --- | --- | --- |
| Ischemic stroke | Ontario Stroke Registry (OSR) | Discharge diagnosis | FD_StrokeYes=Ischemic |
| Estimated glomerular filtration rate (eGFR) | OSR | First serum creatinine value document (usually in the emergency department). Converted to eGFR using the CKD-EPI equation. | EI_CREAT |
| Maintenance dialysis | Canadian Organ Replacement Register (CORR) |  | Recipient_Treatment  Treatment_Code ≠ 171, 181, Transfer_Code ≠ - “W”  Treatment_Date |
| Ischemic stroke etiology | OSR | Categorized as large artery atherosclerosis, cardioembolic, lacunar or other (dissection, prothrombotic state, cortical vein/sinus thrombosis, vasculitis, other). | FD_Ischemic |

**Supplemental Table 3.** Reporting of studies Conducted using Observational Routinely collected health Data (RECORD) statement checklist

|  | **Item No** | **STROBE items** | **RECORD items** | **Reported** |
| --- | --- | --- | --- | --- |
| **Title and abstract** | 1 | (a) Indicate the study's design with a commonly used term in the title or the abstract.  (b) Provide in the abstract an informative and balanced summary of what was done and what was found. | (1.1) The type of data used should be specified in the title or abstract. When possible, the name of the databases used should be included.  (1.2) If applicable, the geographic region and time frame within which the study took place should be reported in the title or abstract.  (1.3) If linkage between databases was conducted for the study, this should be clearly stated in the title or abstract. | Title page |
| **Introduction** |  |  |  |  |
| Background/ rationale | 2 | Explain the scientific background and rationale for the investigation being reported. |  | Introduction |
| Objectives | 3 | State specific objectives, including any prespecified hypotheses. |  | Introduction |
| **Methods** |  |  |  |  |
| Study design | 4 | Present key elements of study design early in the paper. |  | Methods |
| Setting | 5 | Describe the setting, locations, and relevant dates, including periods of recruitment, exposure, follow-up, and data collection. |  | Methods |
| Patients | 6 | (a) Give the eligibility criteria, and the sources and methods of selection of patients. Describe methods of follow-up.  (b) For matched studies, give matching criteria and number of exposed and unexposed. | (6.1) The methods of study population selection (such as codes or algorithms used to identify subjects) should be listed in detail. If this is not possible, an explanation should be provided.  (6.2) Any validation studies of the codes or algorithms used to select the population should be referenced. If validation was conducted for this study and not published elsewhere, detailed methods and results should be provided.  (6.3) If the study involved linkage of databases, consider use of a flow diagram or other graphical display to demonstrate the data linkage process, including the number of individuals with linked data at each stage. | Methods |
| Variables | 7 | Clearly define all outcomes, exposures, predictors, potential confounders, and effect modifiers. Give diagnostic criteria, if applicable. | (7.1) A complete list of codes and algorithms used to classify exposures, outcomes, confounders, and effect modifiers should be provided. If these cannot be reported, an explanation should be provided. | Methods |
| Data sources/   measurement | 8 | For each variable of interest, give sources of data and details of methods of assessment (measurement). Describe comparability of assessment methods if there is more than one group. |  | Methods |
| Bias | 9 | Describe any efforts to address potential sources of bias. |  | Methods |
| Study size | 10 | Explain how the study size was arrived at. |  | Methods/eFigure 1 |
| Quantitative variables | 11 | Explain how quantitative variables were handled in the analyses. If applicable, describe which groupings were chosen and why. |  | Methods |
| Statistical methods | 12 | (a) Describe all statistical methods, including those used to control for confounding.  (b) Describe any methods used to examine subgroups and interactions.  (c) Explain how missing data were addressed.  (d) If applicable, explain how loss to follow-up was addressed.  (e) Describe any sensitivity analyses. |  | Methods |
| Data access and cleaning methods |  | N/A | (12.1) Authors should describe the extent to which the investigators had access to the database population used to create the study population.  (12.2) Authors should provide information on the data cleaning methods used in the study. | Methods |
| Linkage |  | N/A | (12.3) State whether the study included person-level, institutional-level, or other data linkage across two or more databases. The methods of linkage and methods of linkage quality evaluation should be provided. | Methods |
| **Results** |  |  |  |  |
| Patients | 13 | (a) Report numbers of individuals at each stage of study--e.g., numbers potentially eligible, examined for eligibility, confirmed eligible, included in the study, completing follow-up, and analyzed.  (b) Give reasons for non-participation at each stage.  (c) Consider use of a flow diagram. | (13.1) Describe in detail the selection of the persons included in the study (i.e., study population selection), including filtering based on data quality, data availability, and linkage. The selection of included persons can be described in the text and/or by means of the study flow diagram. | Results |
| Descriptive data | 14 | (a) Give characteristics of study patients (e.g., demographic, clinical, social) and information on exposures and potential confounders.  (b) Indicate number of patients with missing data for each variable of interest.  (c) Summarize follow-up time (e.g., average, and total amount). |  | Results |
| Outcome data | 15 | Report numbers of outcome events or summary measures over time. |  | Results |
| Main results | 16 | (a) Give unadjusted estimates and, if applicable, confounder-adjusted estimates and their precision (e.g., 95% confidence interval). Make clear which confounders were adjusted for and why they were included.  (b) Report category boundaries when continuous variables were categorized.  (c) If relevant, consider translating estimates of relative risk into absolute risk for a meaningful time. |  | Results |
| Other analyses | 17 | Report other analyses done (e.g., analyses of subgroups and interactions, and sensitivity analyses). |  | Results |
| Key results | 18 | Summarize key results with reference to study objectives. |  | Results |
| Limitations | 19 | Discuss limitations of the study, considering sources of potential bias or imprecision. Discuss both direction and magnitude of any potential bias. | (19.1) Discuss the implications of using data that were not created or collected to answer the specific research question(s). Include discussion of misclassification bias, unmeasured confounding, missing data, and changing eligibility over time, as they pertain to the study being reported. | Discussion |
| Interpretation | 20 | Give a cautious overall interpretation of results considering objectives, limitations, multiplicity of analyses, results from similar studies, and other relevant evidence. |  | Discussion |
| Generalizability | 21 | Discuss the generalizability (external validity) of the study results. |  | Discussion |
| **Other information** | |  |  |  |
| Funding | 22 | Give the source of funding and the role of the funders for the present study and, if applicable, for the original study on which the present article is based. |  |  |
| Accessibility of protocol, raw data, and programming code |  | N/A | (22.1) Authors should provide information on how to access any supplemental information such as the study protocol, raw data, or programming code. | Acknowledgment |

| **TOAST Subtype** | **Description** |
| --- | --- |
| **Large artery atherosclerosis** | This subtype is characterized by significant narrowing or blockage of major brain arteries such as the internal carotid artery, middle cerebral artery, or vertebral artery. According to the TOAST criteria, significant narrowing is defined as a lumen diameter reduction of 50% or more. This classification is based on imaging studies such as ultrasound, angiography, or MRI. |
| **Cardioembolic** | This subtype is characterized by the presence of a cardiac source that can cause embolism, such as atrial fibrillation, valvular heart disease, or a recent myocardial infarction. The TOAST criteria specify that the cardiac source of embolism should be identified through clinical and laboratory evaluation, which may include electrocardiography, echocardiography, or cardiac biomarkers. |
| **Small vessel disease** | This subtype is characterized by the presence of small infarcts in deep brain regions such as the basal ganglia, thalamus, or brainstem. It is often associated with conditions like hypertension or diabetes. The TOAST criteria define small vessel occlusion as infarcts smaller than 1.5 cm in diameter observed on brain imaging, without significant narrowing or embolism in the large arteries. |
| **Other undetermined etiology** | This subtype is characterized by the presence of other specific causes of stroke, such as arterial dissection, vasculitis, or hypercoagulable states. The TOAST criteria require identification of the underlying cause through clinical evaluation, laboratory testing, or imaging studies, depending on the suspected cause. |
| **Undetermined etiology** | This subtype is used when the cause of the stroke cannot be determined despite a comprehensive evaluation. The TOAST criteria define undetermined etiology as a situation where no specific cause can be identified based on available clinical, laboratory, and imaging data. |

**Supplemental Table 4.** Description of TOAST Classification
